# Factors influencing immunization record retrieval from immunization information systems among independent community pharmacies: A National Survey

**DOI:** 10.1016/j.jvacx.2025.100608

**Published:** 2025-01-16

**Authors:** Oluchukwu M. Ezeala, Nicholas P. McCormick, Christopher L. Meininger, Hannah Fish, John Beckner, David Ha, Rebecca Snead, Heather Vance, Salisa C. Westrick

**Affiliations:** a Auburn University Harrison College of Pharmacy, 4306 Walker Building, Auburn, AL, 36849, USA; b National Community Pharmacists Association, 100 Daingerfield Road Alexandria, VA, 22314, USA; c Stanford Antimicrobial Safety and Sustainability Program, Stanford Health Care, 300 Pasteur Dr, Stanford, CA, 94305, USA

**Keywords:** Pharmacies, Factors, Retrieval, Immunization, Records, IIS

## Abstract

**Background/objectives:**

Community pharmacies may not consistently check patients' immunization records from Immunization Information Systems (IIS) before administering vaccines. Therefore, this study aimed to identify key factors associated with vaccine records retrieval from IIS among community pharmacies.

**Methods:**

A cross-sectional survey of 9446 members of the United States National Community Pharmacists Association was conducted from September–November 2022. There were 492 responses (response rate 5.2 %) received. The analysis included 202 participants who provided immunization services in 2021, administered vaccines besides COVID-19 or influenza, and completed ≥80 % of the questionnaire. The dependent variable was participants' self-reported frequency of retrieving immunization records from IIS (categorized as high or low IIS users). The independent variables considered were participant/pharmacy characteristics, IIS knowledge and perceptions about innovation characteristics, process, and inner settings, as identified by the Consolidated Framework for Implementation Research. Exploratory factor analysis identified ten perception scales, and all had Cronbach's Alpha coefficient of >0.70.

**Results:**

Approximately 65 % of the respondents were high IIS users (“frequently”/“always”) while 35 % were low IIS users (“never”/“rarely”/“occasionally”). A bivariate analyses of the relationship between the dependent and independent variables indicated significant differences in job title, state requirement for documentation in IIS, and eight scales: perceived benefit of IIS in consolidating patient records, optimizing immunization service delivery process, perceived IIS usability, process of engaging staff in IIS implementation, leadership support in encouraging IIS utilization, team values, open communications, and organizational needs fulfillment. A multivariable logistic regression analysis revealed that process optimization (*p* = .009) and leadership support (*p* = .018) were the two significant predictors of being in the high IIS user group when accounting for other variables.

**Conclusion:**

Interventions to encourage independent community pharmacies to retrieve vaccine records from IIS should focus on enhancing the efficiency of the IIS process and leadership support.

## 1 Introduction

Immunization Information Systems (IIS), also known as immunization registries serve as comprehensive databases that collect and consolidate vaccination records for individuals within a given population or geographical area. [1,2] In the United States (U.S.), IIS are publicly run programs. As of 2024, the U.S. has 64 IIS jurisdictions. [3] These comprise all 50 states, certain cities, and some US territories. [3] Each state, local, and territorial authority manages and regulates its own IIS according to its laws and policies, which guide important aspects such as data sharing and provider reporting requirements. [3,4] There is no national IIS in the U.S. currently. [3,5,6] However, the federal government, through the Centers for Disease Control and Prevention (CDC), supports these systems by establishing and promoting IIS functionality standards and providing funding. [4]

IIS offer numerous benefits to public health, healthcare providers and patients. IIS help track immunization coverage, monitor vaccine distribution, and identify potential outbreaks of vaccine-preventable diseases. [7,8] They allow providers quick access to patients' vaccination histories, ensuring timely and appropriate immunizations for their patients. [9] Additionally, IIS serve as valuable resources for patients, offering a centralized platform where they can access their complete vaccination records, promoting health literacy and empowering individuals to take control of their health management. [6,10]

While the benefits of IIS are undeniable, the impact on public health outcomes is dependent upon widespread enrollment and effective utilization by healthcare providers, including community pharmacists. Several initiatives have positively impacted this goal since the introduction of IIS in the 1990's. During the coronavirus disease 2019 (COVID-19) pandemic, the U.S. CDC mandated that pharmacists and other COVID-19 vaccine providers enroll in IIS and report COVID-19 vaccination records to IIS within 72 h of administration. [11] Prior to the pandemic, states such as North Dakota and Oregon also mandated pharmacists to report vaccination records to IIS. [12] In particular, the State of California required pharmacists to report administered vaccines to a California Immunization Registry within 14 days. [13] Researchers also made efforts before the pandemic to increase enrollment of independent community pharmacies in IIS in U.S. states, such as Alabama. [14] These proactive measures are believed to have contributed to a significant increase in enrollment among independent community pharmacies. [15] Meininger and colleagues reported a remarkable rise in independent community pharmacies enrollment in IIS in the U.S., from 65.6 % before the pandemic to nearly 92 % by 2022. [15] The increase in enrollment is particularly important because the role of U.S. community pharmacists in vaccine administration was expanded at the federal level during the COVID-19 pandemic, allowing them to also administer CDC-recommended vaccines to people aged 3–18 years. [[16], [17], [18]] This expansion, combined with their offering of the COVID-19 vaccine, has resulted in a significant rise in the number of other vaccines administered in pharmacy settings. [16] For instance, for U.S. adults, approximately 40–50 % of pneumococcal vaccinations were provided in pharmacies between 2019 and 2022. [16] Additionally, human papillomavirus (HPV) vaccination rates increased by about 12 % by 2022. [19]

Furthermore, independent community pharmacies have shown good performance in reporting vaccination records to IIS alongside the increased enrollment, as Meininger and colleagues reported that, on average, over 80 % of vaccination records for vaccines other than COVID-19 and influenza were reported to IIS by pharmacies in 2021. [15] Besides reporting vaccination records to IIS, immunizers should retrieve vaccination records from IIS prior to vaccine administration to prevent unnecessary vaccination duplications. As described by Lam and colleagues, a regional IIS and health system electronic health record (EHR) in California contained more complete vaccination records than a community pharmacy database, and noted that over 70 % of vaccine recommendations could have changed— possibly preventing inappropriate or unnecessary recommendations—if pharmacists had retrieved and compared these records with their own. [20] This is essentially critical because patients may not be able to accurately recall all their vaccination records, especially for vaccines that are not as frequently administered as those for influenza and COVID-19, such as the tetanus, diphtheria, pertussis (Td/Tdap) vaccines or pneumococcal vaccines. [21] However, Meininger and colleagues reported that a substantial proportion (34.6 %) of pharmacists did not frequently or always retrieve immunization records from IIS before administering vaccines. [15] This lack of consistent retrieval of immunization records before vaccine administration is concerning. [6,20]

In order to design an effective intervention for enhancing the retrieval of vaccine records, it is imperative to first discern the underlying factors contributing to this infrequent behavior. Hastings and colleagues identified factors within four domains—innovation characteristics, process, inner settings, and individual characteristics (specifically knowledge and beliefs about the innovation)—of the Consolidated Framework for Implementation Research (CFIR) that impede the implementation of IIS in the community pharmacy setting through qualitative methods.[22] As such, building on Hastings' study and the CFIR as the study framework, the objective of this study was to identify key factors associated with IIS vaccine records retrieval in independent community pharmacies.

## 2 Methods

### 2.1 Study design, population and setting

This cross-sectional study utilized the online Qualtrics (Qualtrics XM Platform, Provo, Utah) platform to survey 9446 pharmacists, pharmacy owners, and pharmacy technicians who were registered members of the National Community Pharmacists Association (NCPA). NCPA represents all 18,984 independent community pharmacies across the U.S (about 35 % of all community pharmacies in the U.S.). [23] “Independent community pharmacies” here refer to small, pharmacist-owned or privately-owned pharmacies, often located in local communities, that provide specialized healthcare services to their patients. [23,24] All study procedures were approved by the Institutional Review Board of the first author under an exempt protocol.

### 2.2 Data collection

The electronic questionnaire was distributed to NCPA members via 3 email invitations as well as inclusion in 3 of NCPA's electronic newsletters from September 14, 2022, to November 5, 2022. The survey was to be completed by a pharmacist, pharmacy owner, or technician who was knowledgeable about their pharmacy's utilization of their state IIS. To increase response rates, participants who completed the survey were given a chance to participate in a drawing through which they could win one of ten $100 gift cards.

### 2.3 Measures

The main outcome of interest for this study was the frequency with which vaccine records were retrieved from IIS prior to vaccine administration (response options ranged from never [1] to always [5]). The independent variables considered include participant and pharmacy characteristics, as well as the four CFIR domains including the individual knowledge domain and the three perceptions of IIS domains.

Using the questionnaire (Supplementary File 1), participants first shared information about their immunization service provision. Those who offered immunization services in 2021 were asked to select all of the vaccines they provided from a list of CDC-recommended vaccines. Second, participants were asked whether their pharmacy was enrolled in IIS (yes/no/unsure) and if so, they provided information regarding the frequency with which they utilized IIS for retrieval of vaccine records. As for independent variables, we first assessed the first domain – the knowledge of IIS using six items (e.g. “pharmacies can record administered vaccinations in IIS”; true/false/unsure), followed by participant perceptions of IIS in relation to innovation characteristics, process, and inner settings domains, as identified by the CFIR. Specifically, innovation characteristics was evaluated via 25 statements rated using a 7-point Likert scale that ranged from strongly disagree (1) – strongly agree (7). E.g., “using IIS is too time consuming” and “interacting with the IIS is clear and understandable”. Then, perceptions related to 5 statements on the processes of utilizing IIS (e.g., “staff leaders are supportive of the immunization information system”), and 18 statements on inner settings regarding pharmacy personnel involved in immunization services and the use of IIS (e.g., “all staff work together as a team when we implement the immunization information system”) were also assessed. These knowledge and perception statements were all obtained from Hastings' study, which assessed the barriers to implementation of IIS in the community pharmacy setting. [25] However, one knowledge statement, “the Federal government maintains the National Immunization Information System” was adapted from Hasting's original statement,“each state maintains its own Immunization Information System”.

Further, individual demographic and pharmacy characteristics were assessed using both multiple-choice and closed-ended questions, such as “what is the average prescription volume per day at your primary practice location?” Participants were also asked to indicate whether their state required them to report non-seasonal vaccine records to their state IIS for different groups: children aged 10 or younger, adolescents aged 18 or younger, all individuals receiving non-seasonal vaccinations, or if they were not required or unsure. This question was included because the authors believed that pharmacy personnel's beliefs about whether their states require them to report vaccination records to IIS might be just as important as the mandate itself, as these beliefs could influence their records retrieval behaviors. For analysis, we categorized the variable as a binary outcome: participants' responses were coded as “yes” if they stated they were required to report, regardless of the age group, and “no” if they stated they were not required or unsure. The questionnaire was developed by the authors, who ensured its quality and clarity by having it reviewed by three pharmacists before it was launched.

### 2.4 Data analysis

All data analyses were performed using IBM SPSS statistics versions 20.00.00 and 29.00.00 at a predetermined significance level of 0.05 for statistical tests. Participants who did not provide immunization services, only administered COVID-19 and influenza vaccines, and/or completed less than 80 % of the questionnaire were excluded from the analysis. Individual and participant characteristics, knowledge, as well as the dependent variable— frequency of vaccine records retrieval—were summarized using descriptive statistics such as mean, standard deviation, frequency, and percentage as needed. Participants were divided into two major groups based on their IIS record retrieval frequency: high (always, frequently retrieve) and low (never, rarely, and occasionally retrieve) users. Potential non-response bias investigation was conducted by comparing the responses of the first 15 % of respondents to those of the last 15 % using chi-square and Fisher exact tests. [15]

Exploratory factor analysis (EFA) was performed on innovation characteristics, process, and inner settings scales utilizing principal axis factoring (PAF) and varimax rotation with Kaiser normalization to assess the existence and validity of constructs in the scales. Factors with eigenvalues ≥1.0 were retained, and scale items with factor loadings ≥0.400 were included in the analysis. [26,27] EFA of the innovation characteristics scale (Supplementary Table 1) initially revealed 6 factors. However, one factor containing items (2) “checking the pharmacy dispensing software is sufficient to determine immunization status”, (14) “it is difficult to obtain patient consent for IIS”, and (15) “IIS compromise patient confidentiality” was dropped due to poor reliability (Cronbach's alpha <0.700). Additionally, item (22) “I've had the opportunity to test various applications of the IIS” was removed from the “usability of IIS” factor, resulting in improved reliability from 0.692 to 0.701. Items (16) “the IIS appears to have more advantages than disadvantages”, (17) “using the IIS is more reliable than patient self-report when checking immunization status”, (21) “use of the IIS can be adapted to fit our pharmacy's current situation”, and (25) “implementing the IIS is too much of a financial burden” were excluded due to cross-loading into other factors. The final number of factors obtained for the innovation characteristics domain was five, namely: “feasibility of IIS”, “patient immunization records consolidation”, “process optimization”, “usability of IIS”, and “data quality.” The appropriateness for factor analysis within the innovative characteristics domain was deemed adequate, with a Kaiser-Meyer-Olkin MSA (KMO) of 0.859 and Bartlett's Test of Sphericity: χ^2^ = 2137.07 (*p* < .001). Next, EFA was conducted on the process scale (Supplementary Table 2), resulting in the extraction of only one factor called “process engagement”. The factor analysis demonstrated adequacy, with a KMO of 0.770 and Bartlett's Test of Sphericity: χ^2^ = 463.29 (*p* < .001). Lastly, EFA of the inner settings scale yielded 4 factors (Supplementary Table 3). However, items (4) “our pharmacy has proven that we are able to adapt ideas from outside to fit our organization's way of doing things”, (13) “staff incentives are likely to be set up to engage them in using the immunization information system”, (14) “successful implementation of the immunization information system helps us meet our organization's mission and goals”, (17) “our staff do not have the time required to update the immunization information system when an immunization is provided”, and (18) “our staff has access to immunization information system training and training materials” were excluded due to cross-loading into other factors. The resulting four factors were: “leadership support”, “team values”, “open communications”, and “organizational needs fulfillment”. The factor analysis also demonstrated adequacy, with a Kaiser-Meyer-Olkin MSA (KMO) of 0.917 and Bartlett's Test of Sphericity: 2057.48 (*p* < .001). The internal consistency of the 10 perception scales was evaluated using Cronbach's alpha and all had acceptable coefficients of ≥ 0.700 (Supplementary Table 4). We also checked for multicollinearity among the factors (knowledge and the 10 perception scales). The tolerance values varied between 0.374 and 0.951, and the variance inflation factor (VIF) ranged from 1.052 to 2.667. These results suggest that there is no significant multicollinearity present, as VIF values >5 to 10 or tolerance values close to 0 typically indicate significant multicollinearity. [28]

Next, the independent variables were compared between high and low frequency IIS users using bivariate analyses, e.g., one-way ANOVA, Chi-square, and Fisher's exact tests, as appropriate. Subsequently, a multivariable logistic regression analysis was performed using significant variables identified in the bivariate analyses for the frequency of retrieval of vaccination record outcomes.

## 3 Results

Out of the 9446 NCPA members surveyed, 492 responded (response rate = 5.2 %). The margin of error was 7 % at a confidence level of 95 %. The analysis included 202 responses that met the aforementioned inclusion criteria. Except for two characteristics: job title and average pharmacy full-time equivalents (FTEs), the investigation into potential non-response bias revealed no significant differences in characteristics between early and late responders. Notably, as shown in a separate published paper, managers made up a greater percentage of early responders (76.7 %) than late responders (46.7 %) (*p* = .017). [15] On the other hand, early responders had a lower average pharmacy FTEs of 1.8 compared to 2.7 among the late responders' pharmacies (*p* = .010). [15]

Table 1 presents an overview of the participant and pharmacy characteristics. The majority of participants (87.1 %) identified as White, with males accounting for less than half of the total (46.8 %). Nearly half (48.2 %) were between the ages of 41 and 60, while the lowest percentage (18.1 %) were in the 61 to 80 age group. Approximately 91 % of the participants were pharmacists and a higher proportion of them had PharmD (55.4 %). Three-quarters (75.1 %) of the respondents held managerial positions. Very few participants (13.9 %) practiced in non-standalone independent pharmacy settings. PioneerRX® was the most widely used pharmacy software vendor (42.6 %), and slightly more than half (52 %) of respondents' vendors had a feature allowing automatic reporting of vaccination records to IIS. Approximately two-thirds (65.1 %) of pharmacies were located in urban areas.

**Table 1:** Participant and Pharmacy Characteristics.

**Variable**	**Mean (SD)**
Daily prescription volume	*N =* 197 287.60 (239.73)
Full-time equivalents (40 h/1 week) of pharmacists and pharmacy managers	*N =* 193 2.26 (1.37)
	**n (%)**
Age	*N =* 199
22–40	67 (33.7)
41–60	96 (48.2)
61–80	36 (18.1)
Sex	*N =* 201
Female	107 (53.2)
Male	94 (46.8)
Race	*N =* 201
White	175 (87.1)
Non-White	26 (12.9)
Education	*N =* 202
PharmD	112 (55.4)
Non-PharmD	90 (44.6)
Professional title	*N =* 201
Pharmacists	182 (90.5)
Non-Pharmacists	19 (9.5)
Job title	*N =* 197
Manager	148 (75.1)
Non-manager	49 (24.9)
Pharmacy location	*N =* 202
Standalone independent pharmacy	174 (86.1)
Not stand-alone independent pharmacy	28 (13.9)
Pharmacy software vendor	*N =* 197
PioneerRx	84 (42.6)
Transaction Data Systems (e.g., Rx30, Computer-Rx)	58 (29.4)
Other (e.g., Connexus, QS/1)	55 (27.9)
Enrollment in IIS	(*N* = 202)
Yes	185 (91.6)
No	6 (3.0)
Unsure	11 (5.4)
Mechanism of Reporting to IIS	*N = 173*
Automatic	90 (52.0)
Manual	83 (48.0)
Residence^a^	*N = 195*
Metro (1–3)	127 (65.1)
Non-Metro (4–10)	68 (34.9)

a Coding was determined by the USDA's 2010 Rural-Urban Commuting Area (RUCA) codes, with 1 = most urban and 10 = most rural.

Table 2 shows the summary statistic for the dependent variable, “whether participants were high or low IIS users based on their frequency of immunization records retrieval”. Around two-thirds of the participants (65.4 %) reported that they frequently or always retrieved immunization records from IIS and were classified as “high IIS users”. Participants' knowledge of IIS, as shown in Supplementary Table 4, was somewhat unremarkable, just slightly above average with a mean score of 3.62 (± 1.39) out of a total possible score of 6.

**Table 2:** Extent of Records Retrieval from Immunization Information Systems (IIS) for Vaccines Beyond COVID-19 and Influenza Vaccines Among the Study Participants (*N* = 185).

**Variable**	**n (%)**
Frequency in which vaccine records were retrieved from IIS^a^	
Frequently – Always (high users)	121 (65.4)
Occasionally – Never (low users)	64 (34.6)

^a^Response categories ranged from Never (1) to Always (5).

Bivariate analyses of the dependent variable, and participant and pharmacy characteristics (Table 3) revealed significant differences in the variables of job title (*p* = .049) and participants' self-reported state requirement to report vaccine records to IIS (*p* = .009). More specifically, a greater proportion of “high users” of IIS for vaccine records retrieval were mostly pharmacy managers (82.2 %) and those that reported being mandated by their state to report vaccines besides COVID-19 and influenza to their state IIS (77.7 %). For knowledge (Table 4), the bivariate analyses showed no significant difference between the groups for the dependent variable. Additionally, bivariate analyses between retrieval and the 10 perception scales found significant differences in eight of the scales (Table 4), covering the perceived benefit of IIS in terms of consolidating patient records (*p* = .009**)**, optimizing the immunization service delivery process (*p* < .001), perceived IIS usability (*p* = .034), the process of engaging staff in IIS implementation (*p* = .011), leadership support in encouraging IIS utilization (*p* < .001), team values (*p* = .014), open communications (*p* < .001), and organizational needs fulfillment (*p* < .001).

**Table 3:** Bivariate Relationship between Participant and Pharmacy Characteristics and the Frequency of Retrieving Vaccine Records from Immunization Information Systems (IIS) for Vaccines Beyond COVID-19 and Influenza Vaccines (*N* = 185).

**Variable**	**Low Frequency User (occasionally/rarely/never)** **n, Mean (SD)**	**High Frequency User (frequently/always)** **n, Mean (SD)**	**Significance level**
Daily prescription volume^a^	*n* = 63, 244.3 (145.2)	*n* = 118, 310.2 (282.5)	*p* > .05
Full-time equivalents (40 h/1 week) of pharmacists and pharmacy managers^a^	*n =* 62, 2.2 (1.3)	*n =* 117 2.4 (1.4)	*p* > .05
	**n (%)**	**n (%)**	
Age^b^			*p* > .05
22–40	21 (33.3)	37 (30.8)	
41–60	27 (42.9)	63 (52.5)	
61–80	15 (23.8)	20 (16.7)	
Sex^b^			*p* > .05
Female	33 (51.6)	61 (50.4)	
Male	31 (48.4)	60 (49.6)	
Race^b^			*p* > .05
White	56 (87.5)	107 (88.4)	
Non-White	8 (12.5)	14 (11.6)	
Education^b^			*p* > .05
PharmD	38 (59.4)	69 (57.0)	
Non-PharmD	26 (40.6)	52 (43.0)	
Professional Title^c^			*p* > .05
Pharmacists	57 (90.5)	113 (93.4)	
Non-Pharmacists	6 (9.5)	8 (6.6)	
Job title^b^			*p* = .049*
Manager	43 (69.4)	97 (82.2)	
Non manager	19 (30.6)	21 (17.8)	
Reported mandatory requirement by the state to report non-COVID/non influenza vaccine records to IIS^b^			*p* = .009*
Yes	38 (59.4)	94 (77.7)	
No	26 (40.6)	27 (22.3)	
Pharmacy location^b^			*p* > .05
Standalone independent pharmacy	55 (85.9)	107 (88.4)	
Not stand-alone independent pharmacy	9 (14.1)	14 (11.6)	
Pharmacy software vendor^b^			*p* > .05
PioneerRx	31 (50.0)	48 (40.7)	
Transaction Data Systems (e.g., Rx30, Computer-Rx)	18 (29.0)	37 (31.4)	
Other (e.g., Connexus, QS/1)	13 (21.0)	33 (28.0)	
Mechanism of Reporting to IIS^b^			*p* > .05
Automatic	29 (52.7)	60 (51.7)	
Manual	26 (47.3)	56 (48.3)	
Residence^b, d^			*p* > .05
Metro (1–3)	43 (69.4)	78 (66.1)	
Non-Metro (4–10)	19 (30.6)	40 (33.9)	

a One-way ANOVA; ^b^Chi-square; ^c^Fisher Exact; ^d^Coding was determined by the USDA's 2010 Rural-Urban Commuting Area (RUCA) codes, with 1 representing the most urban areas and 10 being the most rural areas

**Table 4:** Bivariate Relationship between Knowledge of Immunization Information Systems (IIS), Innovation Characteristics, Process, Inner Settings Domains and the Frequency of Retrieving Vaccine Records from IIS for Vaccines Beyond COVID-19 and Influenza Vaccines (*N* = 185).

**Factor**	**Low frequency user (occasionally/rarely/never)** **n, mean (SD)**	**High frequency user (frequently/always)** **n, mean (SD)**	**Significance level**^a^
**Individual characteristics domain**			
Knowledge of IIS	*n* = 64, 3.7 (1.5)	*n* = 121, 3.6 (1.4)	*p* > .05
**Innovation characteristics domain**			
Feasibility of IIS	*n =* 64, 4.3 (1.2)	*n =* 121 4.7 (1.3)	*p* > .05
Patient immunization records consolidation	*n =* 64, 5.4 (1.1)	*n =* 121 5.8 (0.9)	*p* = .009*
Process optimization	*n =* 64, 5.0 (1.1)	*n =* 121 5.9 (1.1)	*p* < .001*
Usability of IIS	*n =* 64, 4.2 (1.1)	*n =* 121, 4.6 (1.2)	*p* = .034*
Data quality	*n =* 64, 4.1 (1.0)	*n =* 121, 3.9 (1.3)	*p* > .05
**Process domain**			
Process engagement	*n =* 64, 4.9 (1.0)	*n =* 121, 5.3 (1.0)	*p* = .011*
**Inner settings domain**			
Leadership support	*n =* 63, 5.1 (1.3)	*n =* 119, 5.8 (1.0)	*p* < .001*
Team values	*n =* 63, 5.4 (1.0)	*n =* 119, 5.8 (0.9)	*p* = .014*
Open communications	*n =* 63, 5.1 (1.1)	*n =* 120, 5.7 (1.0)	*p* < .001*
Organizational needs fulfillment	*n =* 63, 5.1 (1.0)	*n =* 119, 5.7 (1.0)	*p* < .001*

a One-way ANOVA; *p < .05.

Table 5 displays the results of the multivariable logistic regression analysis, which included all the significant factors identified in the bivariate comparisons of the frequency of vaccine records retrieval against the independent variables. The analysis revealed that only process optimization (p = .009), from the CFIR innovation characteristics scale, and leadership support (*p* = .018), from the CFIR inner settings scale, significantly increased the odds of being in the “high” IIS user group, suggesting that process optimization and leadership support had a favorable effect on the frequency of immunization record retrieval.

**Table 5:** A Multivariable Logistic Regression Analysis of Factors Associated with Being a High-Frequency User of Immunization Information Systems (IIS) to Retrieve Vaccine Records for Vaccines Beyond COVID-19 and Influenza Vaccines (*N* = 178).

**Factor**	**Exp (B)**	**Significance level**	**95 % C.I. for EXP(B)**	
			**Lower**	**Upper**
**Innovation characteristics domain**				
Patient immunization records consolidation	0.999	0.996	0.648	1.540
Process optimization	1.659	0.009*	1.136	2.423
Usability of IIS	0.959	0.832	0.650	1.415
**Process domain**				
Process engagement	0.856	0.534	0.525	1.396
**Inner settings domain**				
Leadership support	1.869	0.018*	1.111	3.143
Team values	0.729	0.286	0.408	1.303
Open communications	1.173	0.487	0.748	1.842
Organizational needs fulfillment	1.200	0.464	0.736	1.955
**Individual demographic and pharmacy characteristics**				
Job title^a^	0.738	0.491	0.310	1.755
Reported mandatory requirement by the state to report non-COVID/non influenza vaccine records to IIS^b^	0.567	0.157	0.258	1.244
**Constant**	0.012	0.003		

a reference category is manager; ^b^reference category is yes; * *p* < .05.

## 4 Discussions

The study revealed that knowledge of IIS was not a significant predictor of IIS utilization for vaccination records retrieval among the study population. However, it is worth noting that the study found a somewhat low mean score of 3.62 out of 6 for knowledge among the study population, highlighting a gap in understanding among pharmacy personnel regarding IIS operations, management and the various state-specific requirements. Prior studies have demonstrated the importance of IIS knowledge and training to enable implementation in pharmacies. [22,25] While the training as well as the CDC's mandated COVID-19 vaccination reporting requirement to state IIS for all COVID-19 vaccination providers, [11] may have increased pharmacists' knowledge of IIS, our findings show that knowledge of IIS is still insufficient among the study population. This may require additional intervention for improvement on a national scale, which could lead to better and more confident utilization of IIS. Nevertheless, our findings suggest that factors other than knowledge have a greater influence on the national utilization of IIS for retrieving vaccination records before vaccine administration among independent pharmacies. This implies that, while knowledge may be necessary, it is not enough to drive widespread IIS utilization for retrieval purposes.

As stated earlier, different jurisdictions operate their own IIS according to their specific policies. These policies vary in several ways: the type of demographic data required, the types of vaccines reported, the age groups (children or adults) for which reporting is mandatory, and the specific provider types required to report (whether at least one or all). [6] For instance, as of 2021 (the year of interest in this study), Heersema and colleagues noted that, according to state laws at that time, pharmacists and optometrists in California must report pediatric vaccinations to the state IIS. [6] Also, Illinois state law mandates [6] that all active Vaccines for Children (VFC) providers, including dentists, report administered vaccinations to pediatric populations to the state IIS. Regarding adult vaccinations, nineteen states and Philadelphia required every provider to report adult vaccinations to the state IIS, whereas six states and the District of Columbia required only pharmacists to report this data. [6] For pediatric vaccinations, New York City, Philadelphia, and twenty-eight states mandated that all providers report pediatric vaccinations to IIS, while only six jurisdictions required pharmacists to report them. [6] The bivariate result revealed a significant difference (*p* = .009) between high and low-frequency retrievers of vaccine records from IIS based on whether they were required by their states to report vaccine records to IIS. Approximately 78 % of high-frequency retrievers stated that they were mandated to report to the IIS, compared to 59 % of low-frequency retrievers (Table 3). This significant difference suggests that community pharmacies in states with mandated reporting may be more likely to retrieve vaccine records. This could be because knowing (or believing) that they are required to report vaccination records to the IIS likely increases pharmacists' diligence in retrieving these records before administering vaccines. This mandate can improve the perceived reliability and comprehensiveness of IIS data, encouraging pharmacists to use the system more thoroughly to ensure accurate and complete patient vaccination records. The finding supports the American Immunization Registry Association's (AIRA) recommendation for key stakeholders, including State Boards of Pharmacy, the American Pharmacists Association (APhA), the Association of Immunization Managers (AIM), the Association of State and Territorial Health Officials (ASTHO), and the National Association of County and City Health Officials (NACCHO), to advocate for the standardization of state-mandated reporting requirements across U.S. states. Such policies can enhance overall functionality and integration of IIS into pharmacy vaccination practices. [12]

Our findings also highlight the critical influences of job roles, leadership support, and process optimization on the frequency of using IIS to retrieve vaccination records for vaccines beyond COVID-19 and influenza vaccines in the pharmacy setting. Bivariate analyses indicated that managers use IIS more frequently than non-managers, suggesting that managerial or administrative (in some cases) responsibilities and behaviors may promote more consistent IIS use for vaccine records retrieval. This distinction emphasizes the importance of understanding how pharmacy-based job roles and expectations can drive engagement with health information systems. Additionally, our multivariable logistic regression model revealed that leadership support and process optimization significantly influence IIS usage, even when controlling for other variables. Pharmacies where leaders prioritize the success of IIS, value and support its innovative and creative solutions to enhance patient care, and proactively address implementation challenges create an environment conducive to frequent IIS utilization. Such support ensures that staff are motivated and equipped to integrate IIS into their daily workflow, enhancing the overall efficiency and effectiveness of immunization-related activities. Stakeholders seeking to encourage innovation adoption or conduct vaccine education and promotion in pharmacies should engage with pharmacy leadership, as they have been identified as key facilitators in driving and overseeing the implementation of initiatives within the pharmacy setting. Furthermore, the emergence of process optimization as a crucial factor emphasizes the importance of seamlessly integrating IIS into pharmacy operations to facilitate quicker task completion and improve the management of immunization records and vaccination administration. This underscores the need for streamlined processes that enable pharmacy staff to utilize IIS efficiently, thus facilitating their ability to retrieve vaccination records. Collectively, these findings suggest that enhancing leadership support and optimizing processes, alongside recognizing the impact of managerial roles and state-mandated reporting, can significantly increase the frequency of IIS usage for retrieving non-COVID-19/non-Influenza vaccination records in the pharmacy setting, ultimately improving patient care and enabling more effective pharmacy-based immunization service delivery.

### 4.1 Strengths and limitations

One key strength of our study is that we surveyed independent community pharmacies, allowing us to assess how IIS is utilized in diverse and individualized practices commonly found in local communities. Independent pharmacies often have unique operational procedures, decision-making processes, and personalized patient interactions compared to chain pharmacies, which tend to follow standardized protocols. By surveying this population on a national scale, we have identified specific factors influencing use of IIS in retrieving vaccine records in these community pharmacy settings. This could inform more tailored, widespread and effective interventions for improving IIS utilization for this purpose among the study population. In addition, about 35 % of our respondents practiced in rural settings. Given that rural areas often face challenges such as limited access to healthcare providers and immunization services, and that pharmacists, due to their accessibility and expanded role in immunization, play a critical role in increasing vaccine access in these areas, [29] having a substantial portion of respondents from rural areas allowed us to assess factors related to both rural and urban pharmacies that influence IIS utilization. Improving IIS utilization in rural pharmacies by addressing the factors identified in this study can help bridge critical gaps in vaccine delivery and optimize the role of rural pharmacists in these efforts.

This study had several limitations. First, the study had a small sample size, which may limit the generalizability of the findings to a broader population. Second, because the findings are based on self-reported data, there is a possibility of social desirability or recall bias. As a result, participants may have been misclassified into high or low IIS user frequency groups, and/or there may be other inaccuracies in responses. Third, the sample included only members of the U.S. NCPA, so findings may not be applicable to pharmacies or other users outside this group or country. Fourth, we did not confirm whether pharmacies that indicated ‘yes’ to state-mandated reporting to IIS were required to do so by their respective states, or if those that indicated ‘no’ were not required. However, we believe that their beliefs about their state's requirement will impact their behavior in relation to the outcome studied. Lastly, the factors identified in this study are based solely on the considered independent variables, including participant and pharmacy characteristics and CFIR-identified domains— knowledge, innovation characteristics, process, and inner settings as identified previously by Hastings and colleagues. [22]

## 5 Conclusions

This study identified key factors associated with IIS utilization for vaccination records retrieval among NCPA's independent community pharmacies in the U.S. The findings indicate that leadership support for IIS utilization and positive perceptions of IIS in optimizing immunization service delivery are the two significant predictors of being in the ‘high’ IIS vaccine record retrieval group among the study population, when accounting for other significant bivariate relationships. Therefore, efforts to promote vaccine records retrieval from IIS in U.S. independent community pharmacies should focus on improving the efficiency of the IIS process and fostering leadership support.

## CRediT authorship contribution statement

**Oluchukwu M. Ezeala:** Writing – review & editing, Writing – original draft, Formal analysis, Conceptualization. **Nicholas P. McCormick:** Writing – review & editing, Writing – original draft, Formal analysis, Data curation. **Christopher L. Meininger:** Writing – review & editing, Project administration, Formal analysis. **Hannah Fish:** Writing – review & editing, Funding acquisition, Conceptualization. **John Beckner:** Writing – review & editing, Funding acquisition, Conceptualization. **David Ha:** Writing – review & editing, Conceptualization. **Rebecca Snead:** Writing – review & editing. **Heather Vance:** Writing – review & editing, Writing – original draft. **Salisa C. Westrick:** Writing – review & editing, Writing – original draft, Supervision, Project administration, Funding acquisition, Conceptualization.

## Declaration of competing interest

The authors declare the following financial interests/personal relationships which may be considered as potential competing interests: Salisa C. Westrick reports financial support was provided by Centers for Disease Control and Prevention for Auburn University and the National Community Pharmacists Association and that David Ha serves as a consultant as an infectious disease expert. If there are other authors, they declare that they have no known competing financial interests or personal relationships that could have appeared to influence the work reported in this paper.

## Data Availability

Data will be made available on request.

## Appendix A Supplementary data

Supplementary material 1 **Study questionnaire** Supplementary material 1

Supplementary material 2 Exploratory factor analysis results and characteristics of measures for knowledge, innovation characteristics, process and inner setting domains for Immunization Information Systems Supplementary material 2
