# Supplementary material for: Factors influencing immunization record retrieval from immunization information systems among independent community pharmacies: A National Survey
Source: Vaccine X. 2025 Jan 16;23:100608. doi: 10.1016/j.jvacx.2025.100608 (PMC11788848; doi:10.1016/j.jvacx.2025.100608)
Supplement: Supplementary material 1 Study questionnaire [file mmc1.docx]

**Section I: Immunization Service Provision**

- 1. Did your practice site provide immunization services in 2021?

❒ No 🡺 Please indicate why your pharmacy did not provide immunization services

__________ (free text) then 🡺 End the survey; they aren’t qualified to proceed

❒ Yes

- 1. In 2021, which of the following vaccines did your pharmacy provide to**adult**patients? Check all that apply.

❒ COVID-19

❒ *Haemophilus influenzae* type b (Hib)

❒ Hepatitis A (HepA)

❒ Hepatitis B (HepB)

❒ Human Papillomavirus (HPV)

❒ Influenza

❒ Measles, Mumps and Rubella (MMR)

❒ Meningococcal (MenACWY)

❒ Meningococcal (MenB)

❒ Pneumococcal (PCV13, PCV15, PPSV23, PCV20)

❒ Tetanus containing vaccines

❒ Zoster

❒ Not applicable

- 1. In 2021, which of the following vaccines did your pharmacy provide to**pregnant** women? Check all that apply.

❒ COVID-19

❒ Influenza

❒ Hepatitis B (HepB)

❒ Tdap

❒ Not applicable

- 1. In 2021, which of the following vaccines did your pharmacy provide to**adolescents, aged 11-18 years old**? Check all that apply.

❒ COVID-19

❒ Hepatitis A (HepA)

❒ Hepatitis B (HepB)

❒ Human Papillomavirus (HPV)

❒ Influenza

❒ Measles, Mumps and Rubella (MMR)

❒ Meningococcal (MenACWY)

❒ Meningococcal (MenB)

❒ Poliovirus, inactivated

❒ Tdap

❒ Not applicable

- 1. In 2021, which of the following vaccines did your pharmacy provide to**children, aged 10 years or younger**? Check all that apply.

❒ COVID-19

❒ *Haemophilus influenzae* type b (Hib)

❒ Hepatitis A (HepA)

❒ Hepatitis B (HepB)

❒ Influenza

❒ Measles, Mumps and Rubella (MMR)

❒ Pneumococcal conjugate (PCV13)

❒ Poliovirus, inactivated

❒ Tetanus containing vaccines (DTaP, Tdap)

❒ Varicella

❒ Not applicable

1. In this section please indicate the number of doses of each type of vaccine your pharmacy has provided to any age group in 2021.

| **Type of Vaccine** | **Dose Administered** |
| --- | --- |
| COVID-19 |  |
| Diphtheria, tetanus, pertussis (DTaP) |  |
| Hepatitis A |  |
| Hepatitis B |  |
| Herpes Zoster |  |
| Human Papillomavirus (HPV) |  |
| Influenza |  |
| Measles, Mumps, Rubella (MMR) |  |
| Meningococcal (MenACWY) |  |
| Meningococcal (MenB) |  |
| Pneumococcal polysaccharide (PPSV23) |  |
| Pneumococcal 13-valent conjugate (PCV13) |  |
| Pneumococcal 15-valent conjugate (PCV15) |  |
| Pneumococcal 20-valent conjugate (PCV20) |  |
| Poliovirus, inactivated |  |
| Rotavirus (RV) |  |
| Tetanus/ Diphtheria/ Pertussis (Tdap) |  |
| Tetanus/ Diphtheria (Td) |  |
| Travel vaccines (Yellow fever, typhoid, etc.) |  |
| Varicella |  |
| Other (Specify): |  |

**Section II: IISs Practice, Knowledge and Referrals**

Immunization Information Systems (IISs) are computerized databases that record all immunization doses administered by participating providers to persons residing within a given geopolitical area. Sometimes they are referred to as “immunization registries”.

1. Are you required by your state to submit influenza vaccination administration records to the IIS?

❒ Yes, only for children aged 10 or younger

❒ Yes, only for adolescents aged 18 or younger

❒ Yes for all individuals who received influenza vaccinations

❒ No

❒ Unsure

1. Are you required by your state to submit COVID-19 vaccination administration records to the IIS?

❒ Yes, only for children aged 10 or younger

❒ Yes, only for adolescents aged 18 or younger

❒ Yes for all individuals who received COVID-19 vaccinations

❒ No

❒ Unsure

1. Are you required by your state to submit non-seasonal vaccination administration records to the IIS?

❒ Yes, only for children aged 10 or younger

❒ Yes, only for adolescents aged 18 or younger

❒ Yes for all individuals who received non-seasonal vaccinations

❒ No

❒ Unsure

1. Are your pharmacies enrolled in the immunization information system (IIS)?

❒ Yes 🡺 Go to 5a-e

❒ No 🡺 Go to 5f

❒ Unsure 🡺 Go to 6

1. 5a Of the following, which best describes the year when your pharmacy first enrolled in the IIS?

❒ 2011 or before

❒ 2012-2015

❒ 2016-2019

❒ 2020

❒ 2021

❒ 2022

5b How often do your pharmacy personnel use the IIS to retrieve patients’ immunization history in order to verify the need for non-COVID/non-influenza vaccine(s) before vaccine administration?

❒ Never

❒ Rarely

❒ Occasionally

❒ Frequently

❒ Always

5bb. For those who didn’t answer Never on 5b; what prompt your pharmacy personnel to go into IIS to retrieve patients’ immunization history?

❒ It is part of our routine workflow

❒ It is for new patient

❒ We do it when a patient requests

❒ We do it when a provider requests

❒ We do it when we give multiple vaccines at the same time

❒ Other. Please describe ____________

5c For vaccinations completed at your pharmacy, what percentage of influenza vaccination records were uploaded to the IIS in 2021?

*Have a ruler for them to mark*

5d What percentage of non-COVID/Non-influenza vaccination records were uploaded to the IIS in 2021?

*Have a ruler for them to mark*

5e During the COVID-pandemic, COVID-19 vaccine records must be reported to their respective IIS. Because your pharmacy must adapt your workflow to accommodate this IIS requirement for COVID-19 vaccines, how easy is it for your pharmacy to incorporate the same practice for other vaccines?

❒ Not at all

❒ A little

❒ Somewhat

❒ To a great extent

5f You responded that your pharmacies are not enrolled in the IIS. Please indicate the level of agreement with the following statements. Response categories from 1 (Strongly Disagree) to 7 (Strongly Agree).

❒ I plan to enroll my pharmacy in the IIS within 30 days. ____

❒ I will make an effort to enroll my pharmacy in the immunization information system in the next 30 days. ____

❒ I do not plan intend to enroll my pharmacy in the immunization information system. _____

1. Which of the following describe the vaccination record keeping practices that your pharmacy personnel engage in for vaccinations administered at your pharmacy? Check all that apply.

❒ Giving a receipt for vaccines received at the pharmacy to the patient

❒ Documenting on the patient’s personal immunization record or the patient’s vaccination card and giving it to the patient to keep

❒ Documenting in the immunization information system (IIS) 🡺 How?

❒ Automatic electronic transmission

❒ Manual Upload

❒ Other – Describe:___

❒ Don’t know

❒ Documenting in the pharmacy software 🡺 Does your software link with the EMR/EHR of the patient’s healthcare provider?

❒ Yes

❒ No

❒ Don’t know

❒ Manually reporting to the patient’s primary care provider 🡺 How?

❒ By fax

❒ By hard copy

❒ By phone

❒ Don’t know

❒ Other – Describe:___

❒ Other. Please specify: ________________________________

1. Please indicate whether you believe the following statements are true, false, or if you are unsure.

|  | True | False | Unsure |
| --- | --- | --- | --- |
| 1. Immunization information systems (IISs) consolidate vaccination data for patients within a defined geographic area. | ❒ | ❒ | ❒ |
| 1. Pharmacies can record administered vaccinations in IISs. | ❒ | ❒ | ❒ |
| 1. Other providers can retrieve immunization information from IISs in real time. | ❒ | ❒ | ❒ |
| 1. The Federal government maintains the National Immunization Information System. | ❒ | ❒ | ❒ |
| 1. Immunization information systems consolidate immunization doses from all providers regardless of IIS participation. | ❒ | ❒ | ❒ |
| 1. Immunization information system reporting for community pharmacy is mandatory in all states. | ❒ | ❒ | ❒ |

1. At the pharmacy where you work, when it is necessary, do you or other staff refer patients to another provider or location for vaccination?

❒ Yes 🡺 Does your pharmacy follow up with the patient later to see if they receive the vaccination (Yes/No)

🡺 Does your pharmacy follow up with the provider or the other location to see if they vaccinate the patient (Yes/No)

🡺Where does your pharmacy normally refer patients to

 ❒ Health department

 ❒ Pharmacy/another pharmacy

 ❒ Medical clinic

 ❒ Travel clinic

 ❒ Other : Describe:__________

❒ No

❒ Unsure

1. Where does your pharmacy publicize available vaccines?

❒ On the pharmacy website

❒ On the pharmacy’s social media channels such as facebook, instagram, twitter

❒ Vaccines.gov for COVID-19 vaccine

❒ State or regional website

❒ Other; please specify _____

**Section III: IIS Perceptions**

**Instructions:** Regardless of whether your pharmacy is enrolled in the IIS, please respond how strongly do you agree or disagree with the following statements.

1. How strongly do you agree or disagree with the following statements?

|  | Strongly  Disagree  (1) | (2) | (3) | (4) | (5) | (6) | Strongly  Agree  (7) |
| --- | --- | --- | --- | --- | --- | --- | --- |
| 1. IISs are not standardized in terms of data required. |  |  |  |  |  |  |  |
| 1. Checking the pharmacy dispensing software is sufficient to determine immunization status. |  |  |  |  |  |  |  |
| 1. Data recorded in IISs is inaccurate. |  |  |  |  |  |  |  |
| 1. Data recorded in IISs is incomplete. |  |  |  |  |  |  |  |
| 1. IISs provide patients with consolidated immunization records. |  |  |  |  |  |  |  |
| 1. IISs improve patient care coordination. |  |  |  |  |  |  |  |
| 1. IISs allow pharmacies to assess immunization status. 2. IISs are a realistic method of consolidating immunization data |  |  |  |  |  |  |  |
| 1. Using IISs are too time consuming. |  |  |  |  |  |  |  |
| 1. The IIS fits easily into pharmacy workflow. 2. It is difficult to obtain access to the IIS. 3. It is difficult to obtain software that connects with IIS. 4. It is difficult to obtain IT support for IIS. 5. It is difficult to obtain patient consent for IIS. 6. IISs compromise patient confidentiality 7. The IIS appears to have more advantages than disadvantages. 8. Using the IIS is more reliable than patient self-report when checking immunization status. 9. Using the IIS enables pharmacy staff to accomplish tasks related to the provision of immunizations more quickly. 10. Using the IIS enables pharmacy staff to accomplish tasks related to the provision of immunizations more effectively. 11. The IIS helps to manage vaccine inventory more effectively. 12. Use of the IIS can be adapted to fit our pharmacy’s current situation 13. I’ve had the opportunity to test various applications of the IIS. 14. Interacting with the IIS is clear and understandable. 15. Interacting with the IIS does not require a lot of mental effort. 16. Implementing the IIS is too much of a financial burden. |  |  |  |  |  |  |  |

**For those who are enrolled in IIS** – The following statements are about processes of utilizing IIS. Utilizing IIS consists of both transmitting immunization records when patients receive the vaccinations as well as retrieving patients’ immunization records to determine immunization gaps. Please respond how strongly do you agree or disagree with the following statements.

|  | Strongly  Disagree  (1) | (2) | (3) | (4) | (5) | (6) | Strongly  Agree  (7) |
| --- | --- | --- | --- | --- | --- | --- | --- |
| 1. When implementing the immunization information system, we identify specific roles and responsibilities. |  |  |  |  |  |  |  |
| 1. When implementing the immunization information system, we clearly describe tasks and timelines. |  |  |  |  |  |  |  |
| 1. When implementing the immunization information system, we include appropriate staff education. |  |  |  |  |  |  |  |
| 1. When implementing the immunization information system, we acknowledge staff input and opinions. |  |  |  |  |  |  |  |
| 1. Staff leaders are supportive of the immunization information system. |  |  |  |  |  |  |  |
| 1. The individual responsible for overseeing implementation of the immunization information system is committed to making this successful. |  |  |  |  |  |  |  |
| 1. The majority of our pharmacy staff have been involved in the decision to implement the immunization information system. |  |  |  |  |  |  |  |
| 1. We have spoken with patients and considered their opinion regarding the immunization information system. |  |  |  |  |  |  |  |
| 1. We collect honest reactions from staff regarding use of the immunization information system |  |  |  |  |  |  |  |

**For those who are enrolled in IIS** – The following statements are about pharmacy personnel who may be involved in immunization services and the use of IIS. Please respond how strongly do you agree or disagree with the following statements.

|  | Strongly  Disagree  (1) | (2) | (3) | (4) | (5) | (6) | Strongly  Agree  (7) |
| --- | --- | --- | --- | --- | --- | --- | --- |
| 1. All staff work together as a team when we implement the immunization information system. |  |  |  |  |  |  |  |
| 1. The changes that occurred in the pharmacy when implementing the immunization information system are communicated to all pharmacy staff. |  |  |  |  |  |  |  |
| 1. Mechanisms for communication, such as staff meetings, are important when implementing the immunization information system. |  |  |  |  |  |  |  |
| 1. Our pharmacy has proven that we are able to adapt ideas from outside to fit our organization’s way of doing things. |  |  |  |  |  |  |  |
| 1. Our pharmacy owner/manager rewards innovation and creativity to improve patient care. |  |  |  |  |  |  |  |
| 1. Staff members in our pharmacy have a sense of personal responsibility for improving patient care and outcomes. |  |  |  |  |  |  |  |
| 1. Staff members in our pharmacy cooperate to maintain and improve effectiveness of patient care. |  |  |  |  |  |  |  |
| 1. Staff members in our pharmacy are willing to innovate and/or experiment to improve patient care. |  |  |  |  |  |  |  |
| 1. Staff members in our pharmacy are receptive to change. |  |  |  |  |  |  |  |
| 1. Some of our pharmacy staff believe that implementing the immunization information system is essential. |  |  |  |  |  |  |  |
| 1. Successfully implementing the immunization information system meets staff needs. |  |  |  |  |  |  |  |
| 1. Our pharmacy owner/manager has set a high priority on the success of the immunization information system in our pharmacy. |  |  |  |  |  |  |  |
| 1. Staff incentives are likely to be set up to engage them in using the immunization information system. |  |  |  |  |  |  |  |
| 1. Successful implementation of the immunization information system helps us meet our organization’s mission and goals. |  |  |  |  |  |  |  |
| 1. The pharmacy owner/ manager/ staff opinion leaders agree on the goals for the implementation of the immunization information system. |  |  |  |  |  |  |  |
| 1. Our pharmacy owner/manager has committed to spending time and resources to remove obstacles related to implementation of the IIS if they arise. |  |  |  |  |  |  |  |
| 1. Our staff do not have the time required to update the immunization information system when an immunization is provided. |  |  |  |  |  |  |  |
| 1. Our staff has access to immunization information system training and training materials. |  |  |  |  |  |  |  |

**Section IV: Individual and Pharmacy Characteristics**

1. Please indicate your sex:

❒ Male

❒ Female

1. Please indicate your race:

❒ White

❒ Black or African American

❒ Asian

❒ Native Hawaiian or Other Pacific Islander

❒ American Indian or Alaska Native

❒ Other. Please specify: __________

1. Please indicate your ethnicity:

❒ Hispanic or Latino

❒ Not Hispanic or Latino

1. Please indicate your age: _________ years
2. Please indicate your title. Select all that apply.

❒ Staff pharmacist

❒ Pharmacist pharmacy owner/partner/manager

❒ Non-pharmacist pharmacy owner/partner/manager

❒ Pharmacy technician or clerk

❒ Student pharmacist

❒ Other. Please specify: __________

1. Please indicate your education/training. Select all that apply.

❒ B.S. Pharmacy

❒ PharmD

❒ Pharmacy technician certification

❒ Residency in Pharmacy

❒ Masters of Pharmacy

❒ Other. Please specify: __________

1. Which of the following best describes the pharmacy that is your primary practice location?

❒ Stand-alone independent pharmacy

❒ Pharmacy embedded within a medical clinic or a hospital

❒ Pharmacy within a grocery or retail store

❒ Other. Please specify: __________

1. What is the name of your pharmacy software vendor? ________
2. Please indicate the zip code of your primary practice location: ____________
3. What is the average prescription volume per day at your primary practice location? _____ Prescriptions per day
4. How many FTEs (Full-time equivalents, 40 hrs/wk) of pharmacists, including the pharmacy manager, does your pharmacy schedule, including both PharmD and BS Pharm?

_____ FTEs
