# Supplementary material for: Factors influencing immunization record retrieval from immunization information systems among independent community pharmacies: A National Survey
Source: Vaccine X. 2025 Jan 16;23:100608. doi: 10.1016/j.jvacx.2025.100608 (PMC11788848; doi:10.1016/j.jvacx.2025.100608)
Supplement: Supplementary material 2 Exploratory factor analysis results and characteristics of measures for knowledge, innovation characteristics, process and inner setting domains for Immunization Information Systems [file mmc2.docx]

Supplementary Table 1: Exploratory Factor Analysis of Innovation Characteristics Domain at a Factor

Loading of 0.4 or Greater

|  | **Components (factor loading level @ ≥.40)** | | | | |
| --- | --- | --- | --- | --- | --- |
| **Items^(#)a^** | **1** | **2** | **3** | **4** | **5** |
| **Feasibility of IIS** |  |  |  |  |  |
| Using IISs are too time consuming. (9)^a^ | .563 |  |  |  |  |
| The IIS fits easily into pharmacy workflow. (10) | .623 |  |  |  |  |
| It is difficult to obtain access to the IIS. (11)^a^ | .598 |  |  |  |  |
| It is difficult to obtain software that connects with IIS. (12)^a^ | .810 |  |  |  |  |
| It is difficult to obtain IT support for IIS. (13)^a^ | .647 |  |  |  |  |
| **Patient immunization records consolidation** |  |  |  |  |  |
| IIS provide patients with consolidated immunization records. (5) |  | .605 |  |  |  |
| IIS improve patient care coordination. (6) |  | .730 |  |  |  |
| IIS allow pharmacies to assess immunization status. (7) |  | .627 |  |  |  |
| IISs are a realistic method of consolidating immunization data. (8) |  | .750 |  |  |  |
| **Process optimization** |  |  |  |  |  |
| Using the IIS enables pharmacy staff to accomplish tasks related to the provision of immunizations more quickly. (18.) |  |  | .781 |  |  |
| Using the IIS enables pharmacy staff to accomplish tasks related to the provision of immunizations more effectively. (19) |  |  | .804 |  |  |
| **Usability of IIS** |  |  |  |  |  |
| The IIS helps to manage vaccine inventory more effectively. (20) |  |  |  | .434 |  |
| Interacting with the IIS is clear and understandable. (23) |  |  |  | .821 |  |
| Interacting with the IIS does not require a lot of mental effort. (24) |  |  |  | .630 |  |
| **Data quality** |  |  |  |  |  |
| IISs are not standardized in terms of data required. (1)^a^ |  |  |  |  | .538 |
| Data recorded in IIS is inaccurate (3)^a^ |  |  |  |  | .761 |
| Data recorded in IIS is incomplete. (4)^a^ |  |  |  |  | .696 |

Bartlett’s Test of Sphericity: x^2^ = 2137.07 (p < 0.001); Kaiser-Meyer-Olkin MSA = 0.859. ^(#)^Indicates the corresponding item

numbering in the questionnaire; ^a^Reverse-coded items.

Supplementary Table 2: Exploratory Factor Analysis of Process Domain at a Factor Loading of 0.4 or greater

| **Item** | **Components (factor loading level @ ≥.40)** |
| --- | --- |
| **Process engagement** |  |
| When implementing the immunization information system, we identify specific roles and responsibilities. (1) | .741 |
| When implementing the immunization information system, we clearly describe tasks and timelines. (2) | .781 |
| When implementing the immunization information system, we include appropriate staff education. (3) | .825 |
| When implementing the immunization information system, we acknowledge staff input and opinions. (4) | .721 |
| Staff leaders are supportive of the immunization information system. (5) | .627 |

Bartlett’s Test of Sphericity: x^2^ = 463.29 (p < 0.001); Kaiser-Meyer-Olkin MSA = 0.770.

Supplementary Table 3: Exploratory Factor Analysis of Inner Settings Domain at a Factor Loading

of 0.4 or greater

|  | **Components ((factor loading level @ ≥.40))** | | | |
| --- | --- | --- | --- | --- |
| **Items(#)** | **1** | **2** | **3** | **4** |
| **Leadership support** |  |  |  |  |
| Our pharmacy owner/manager rewards innovation and creativity to improve patient care. (5) | .674 |  |  |  |
| Our pharmacy owner/manager has set a high priority on the success of the immunization information system in our pharmacy. (12) | .762 |  |  |  |
| The pharmacy owner/ manager/ staff opinion leaders agree on the goals for the implementation of the immunization information system. (15) | .645 |  |  |  |
| Our pharmacy owner/manager has committed to spending time and resources to remove obstacles related to implementation of the IIS if they arise. (16) | .685 |  |  |  |
| **Team values** |  |  |  |  |
| Staff members in our pharmacy have a sense of personal responsibility for improving patient care and outcomes. (6) |  | .768 |  |  |
| Staff members in our pharmacy cooperate to maintain and improve effectiveness of patient care. (7) |  | .704 |  |  |
| Staff members in our pharmacy are willing to innovate and/or experiment to improve patient care. (8) |  | .708 |  |  |
| Staff members in our pharmacy are receptive to change. (9) |  | .518 |  |  |
| **Open communications** |  |  |  |  |
| All staff work together as a team when we implement the immunization information system. (1) |  |  | .527 |  |
| The changes that occurred in the pharmacy when implementing the immunization information system are communicated to all pharmacy staff. (2) |  |  | .716 |  |
| Mechanisms for communication, such as staff meetings, are important when implementing the immunization information system. (3) |  |  | .643 |  |
| **Organizational needs fulfilment** |  |  |  |  |
| Some of our pharmacy staff believe that implementing the immunization information system is essential. (10) |  |  |  | .583 |
| Successfully implementing the immunization information system meets staff needs. (11) |  |  |  | .839 |

Bartlett’s Test of Sphericity: x^2^ = 2057.48 (p < 0.001); Kaiser-Meyer-Olkin MSA = 0.917. (#)Indicates the corresponding

item numbering in the questionnaire.

Supplementary Table 4: Characteristics of Measures for Knowledge, Innovation Characteristics,

Process and Inner Settings Domains for Immunization Information Systems (IIS)

| **Factor^a^** | **Number of items** | **Mean (SD)** | **Cronbach’s α** |
| --- | --- | --- | --- |
| **Individual characteristics domain** |  |  |  |
| Knowledge of IIS *(N = 202)* | 6 | 3.62 (1.39) | - |
| **Innovation characteristics domain** |  |  |  |
| Feasibility of IIS *(N = 202)* | 5 | 4.49 (1.26) | .82 |
| Patient immunization records consolidation *(N = 202)* | 4 | 5.62 (1.00) | .83 |
| Process optimization *(N = 202)* | 2 | 5.51 (1.21) | .90 |
| Usability of IIS *(N = 202)* | 3 | 4.40 (1.14) | .70 |
| Data quality *(N = 202)* | 3 | 3.92 (1.24) | .71 |
| **Process domain** |  |  |  |
| Process engagement *(N = 185)* | 5 | 5.15 (1.02) | .86 |
| **Inner settings domain** |  |  |  |
| Leadership support*(N = 182)* | 4 | 5.53 (1.13) | .88 |
| Team values *(N = 182)* | 4 | 5.64 (0.95) | .87 |
| Open communications *(N = 183)* | 3 | 5.46 (1.07) | .78 |
| Organizational needs fulfilment *(N = 182)* | 2 | 5.50 (1.07) | .82 |

^a^Scales ranged from 1 = strongly disagree to 7 = strongly agree.
